# The impact of the titanium cranial hardware in proton single‐field uniform dose plans

**DOI:** 10.1002/acm2.14374

**Published:** 2024-06-12

**Authors:** Peng Wang, Fionnbarr Timothy O'Grady, Martin Janson, Daniel Kim, Kevin S. Choe, MacLennan Grayden, Caroline K. Bamberger, Jiajin Fan

**Affiliations:** ^1^ Advanced Radiation Oncology and Proton Therapy Inova Health System Fairfax Virginia USA; ^2^ RaySearch Laboratories Stockholm Sweden

**Keywords:** cranial titanium hardware, proton beam perturbation, proton Monte Carlo dose calculation, proton SFO planning, proton therapy

## Abstract

**Background:**

Neurosurgical cranial titanium mesh and screws are commonly encountered in postoperative radiation therapy. However, only a limited number of reports are available in the context of proton therapy, resulting in a lack of consensus among the proton centers regarding the protocol for handling the hardware.

**Purpose:**

This study is to examine the impact of the hardware in proton plans. The results serve as evidence for proton centers to generate standard operating procedures to manage the hardware in proton treatment.

**Methods:**

Plans with different gantry angles and material overrides are generated on the CT images of a phantom made of the hardware. The dose distributions of the plans with and without material override, at different depths are compared. Films and ionization chambers are used to measure the plans and the measurements are compared to the treatment planning system (TPS) calculations by gamma analysis.

**Results:**

There are some overdose and underdose regions downstream of the hardware. The overdose and underdose values are within a few percent of the prescribed dose when multiple fields with large hinge angles are used. The gamma analysis results show that the measurements agree with the TPS calculations within limits that are clinically relevant.

**Conclusion:**

The study has demonstrated the influence of the hardware on proton plans. Based on the result of this study, a standard operating procedure of managing the hardware has been implemented in our clinic.

## 1 INTRODUCTION

Dosimetric comparison studies have shown that proton therapy is able to avoid excessive integral radiation dose to a variety of normal structures at all dose levels while maintaining equal target coverage,^1^, ^2^, ^3^, ^4^, ^5^ which may explain the lower neurocognitive side effects associated with proton therapy on pediatric patients.^6^, ^7^, ^8^, ^9^ In addition, a review shows a significant increase in the use of proton therapy for the treatment of adults with primary brain tumors between 2004 and 2015.^10^

Neurosurgical cranial titanium mesh and screws are commonly encountered in postoperative radiation therapy. Extensive studies have been conducted on the dosimetric effects of this hardware in photon and electron therapy.^11^, ^12^, ^13^, ^14^, ^15^ However, only a limited number of reports are available in the context of proton therapy.^16^, ^17^ There is no consensus among the proton centers in the United States regarding the protocol for handling titanium cranial hardware, as shown in a recent survey conducted by the authors of this manuscript (see the Appendix S1). Some centers contour the hardware, create a margin around it, and prevent the proton beams from passing through the region. The advantage of this approach is that it eliminates the uncertainty of the modeling of the hardware in the treatment planning system (TPS).This may necessitate the use of multi‐field optimization (MFO) to achieve adequate coverage, however, this produces treatment plans that are generally less robust than those with uniform dose throughout the target volume from each field (single‐field uniform dose, a.k.a. SFUD)^18^ via single‐field optimization (SFO).^19^ In addition, the low‐dose region around the hardware and margin may not be desirable when the tumor bed is adjacent to the hardware.

In this study, we investigated the dose distribution when proton spots were allowed to pass through the hardware in SFUD plans. The main questions to be answered were:

1. When robust optimization, including isocenter and range uncertainties,^20^ was implemented in planning, was the impact of the hardware on the dose distribution significant enough for the physicists to take special actions on the hardware?
2. Is it clinically necessary to contour and assign a material to the hardware?

## 2 MATERIALS AND METHODS

### 2.1 Phantom and detectors

**FIGURE 1:**
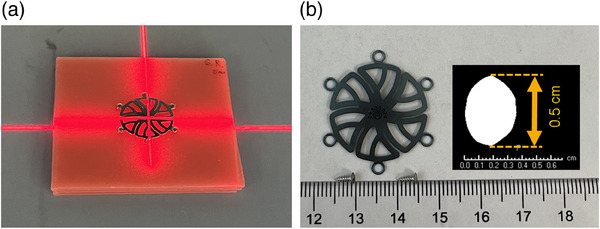
(a) Phantom setup during the plan delivery. (b) A photograph of the mesh and screws. The inset is a CT axial view of one screw with the lowest HU value set to 600.

The thickest mesh and the longest screw from the supplier are used in this study. The mesh and screws (MatrixNEURO) are 0.06 cm thick and 0.50 cm long with diameters of 4.00 cm and 0.15 cm, respectively. The mesh was secured by the screws on a wax cube with a side length of 8.00 cm and a thickness of 0.60 cm. Five screws were used with the most inferior screw hole on the mesh unoccupied. The screws were oriented such that their axes were perpendicular to the top surface of the wax cube. Solid phantom plates (Solid Water HE by SUN NUCLEAR) were placed underneath the phantom during the proton plan delivery, as seen in Figure 1a.

Gafchromic EBT3 films (Ashland, Wilmington, Delaware, USA) were used in the validation studies so that both absolute dose and high‐resolution dose distributions could be obtained simultaneously. To reduce the proton linear energy transfer (LET) effect on film optical density,^21^ the films were calibrated at a similar depth in a similar plan. An optical density versus dose conversion curve was generated and was applied to all the films in this study. Films were placed at different depths inside and outside of the target contour. A PPC05 ionization chamber (IBA Dosimetry, Germany) was used as the secondary dose verification. All proton beams were delivered such that the solid water stack, with the film and ion chamber, were downstream of the phantom containing the hardware.

### 2.2 Proton treatment planning, delivery systems, and CT scanner

The proton delivery system used in this study is the IBA Proteus Plus (Ion Beam Applications, Belgium) multi‐room system with dedicated proton pencil beam scanning. The treatment planning system used in the study is Raystation version 11A (RaySearch Laboratories, Stockholm, Sweden). The computed tomography (CT) scanner used in the study is the GE Revolution with slice thickness of 0.125 cm, pixel size of 0.098 cm and metal artifact reduction (MAR). Gammex Model 467 phantom was used to generate the calibration curve between the Hounsfield Unit (HU) values and mass density.

### 2.3 Treatment planning

The titanium plug in the Gammex phantom is a cylinder with a diameter of 1.3 cm and a length of 7.0 cm. To accurately delineate the plug, the HU threshold value was set to 4000. The range of the HU values of the contour was between 4000 and 9000. However, the above parameters did not apply to the titanium screws. The HU threshold value was set to 600 (Figure 1b), which accurately delineated the length of the screw, but overestimated the diameter of the screw by a factor of two. The HU values of the screws ranged from 600 to 4336. The corresponding ranges of the mass density and relative linear stopping power for a proton energy of 115 MeV are 1.39∼2.98 g/cm^3^ and 1.32∼2.28, respectively. The maximum HU value in our HU‐to‐density table is 3071. The maximum density in the table is assigned to voxels with HU values higher than 3071 if no material override is assigned to them. When Titanium is assigned as the material to a contour, the mass density of 4.54 g/cm^3^ and the relative linear stopping power in the range of 3.18–3.24 are assigned. Meanwhile, the contouring and material assignment of the mesh are not performed in the study. First, the thickness of the mesh is much smaller than the voxel size of the CT image (∼0.10 cm), which leads to inaccurate HU values of metal due to the averaging effect. Second, it is unlikely that the mesh diameter axis is parallel to the proton beam direction in real patient plans.

Two groups of plans were created. In Group I, the hardware was not contoured, and the material was not assigned. The mass density of the hardware was converted from the HU values via the calibration curve. In Group II, the hardware was contoured, and the material was overridden to titanium manually.

The target was defined as a cube with a side length of 5.0 cm at a depth of 6.0 cm. Robust optimization was implemented with an isotropic uncertainty in isocenter position of 0.3 cm and a range uncertainty of 3.5%.^20^ Three plans were generated, each with two anterior oblique fields at beam angles of ± 5°, ± 15°, and ± 30°. Each plan was optimized for a target coverage of V98%Rx = 98% using a single‐field optimization technique. The Monte Carlo dose calculation algorithm was used in both optimization and calculation.

## 3 RESULTS

### 3.1 Dose distribution in the TPS

**FIGURE 2:**
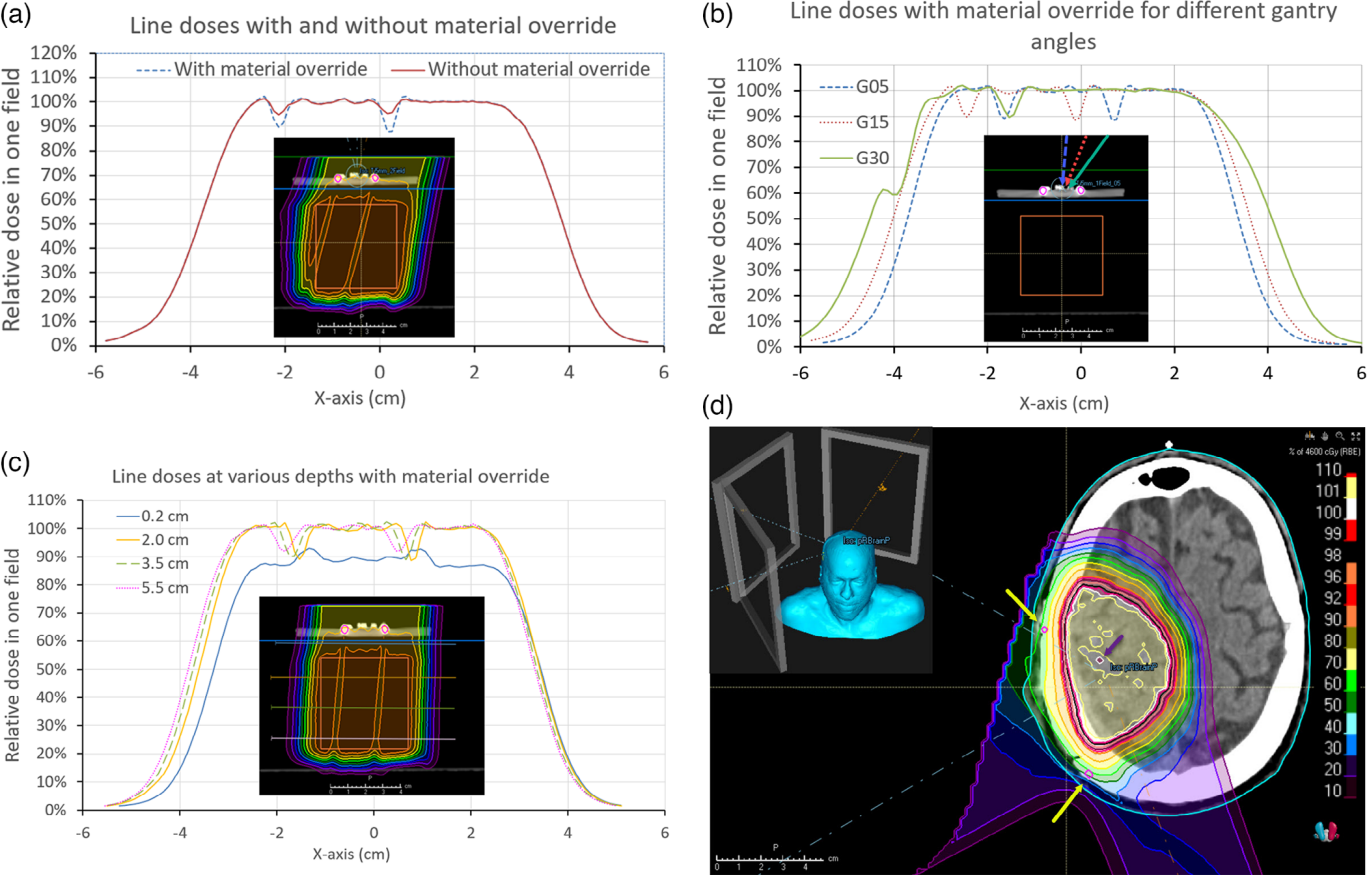
(a) TPS dose profiles in the target region at a depth of 3.5 cm for a gantry angle of 15° with and without the material override. (b) TPS dose profiles at a depth of 3.5 cm for three plans with different gantry angles with the hardware overridden to titanium. (c) TPS dose profiles at four depths for the plan with gantry angle of 5°. (d) A proton treatment plan for meningioma. The yellow arrows indicate the titanium screws with material override. The purple arrow indicates the underdose region.

Figure 2 shows the TPS dose distributions of only one field of all the plans. Figure 2a shows the dose distribution in the target region at a depth of 3.5 cm with and without a material override on the hardware at the gantry angle of 15°. The main difference between the two distributions within the target is the degree of underdosing behind the screws. It is ∼10% and ∼5% of the prescribed dose per field for plans with and without material override, respectively. Figure 2b shows the dose profile at a depth of 3.5 cm with different gantry angles when the hardware was overridden to titanium. The underdoses behind the screws are almost the same for all gantry angles. Figure 2c shows the dose profiles at four depths in the plan with gantry angle of 5°. At the depth of 0.2 cm, the overdose downstream of the screws is approximately 6%. The overdose is about 4% for the plans without material override. Figure 2c also shows that the underdose downstream of the screws changes from ∼11% to ∼8% as the depth changes from 2.0  to 5.5 cm.

### 3.2 Measurements

**FIGURE 3:**
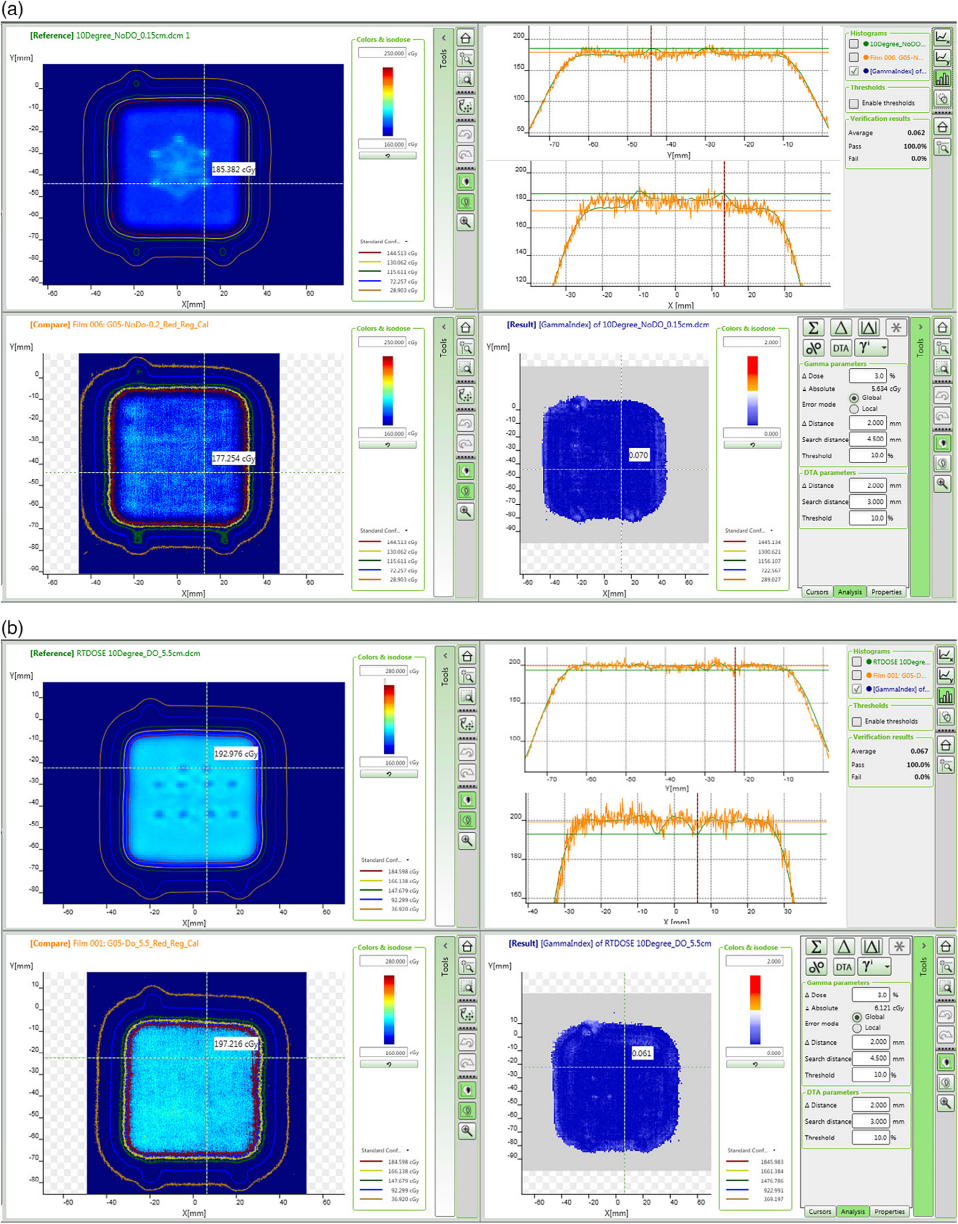
(a) Gamma analysis between the TPS calculations and the film measurement at 0.2 cm in depth in the plan without material overridden. (b) Gamma analysis between the TPS calculations and the film measurement at 5.5 cm in depth in the plan with the hardware overridden to titanium.

For each film measurement, a gamma analysis was performed with our clinical settings, that is, a dose (Δ*D*) and distance (Δ*d*) criteria of 3%/2 mm and a 10% global dose threshold between the measurements and the TPS calculations with and without a density override. The pass rates are all 100% for the plans with and without the material override. Figure 3a shows the analysis on the film placed 0.2 cm below the hardware, in the plan without material override. Figure 3b shows the analysis on the film placed 5.5 cm below the hardware, in the overridden plan.

The delivered dose was measured with an ionization chamber in the center of the target, and the result showed that the difference between the measurement and the TPS calculation was within 1.0%.

## 4 DISCUSSION

For TPS calculations, it is shown that when using robust optimization and multiple fields, “hot” and “cold” regions still exist in the region downstream of the titanium hardware. For the cranial screws of 0.5 cm in length, the overdosage directly below the screws is about 6% and 4% per field for the plans with and without material override, respectively. This overdose becomes an underdose with increasing depth. The underdose inside the target is shown to be approximately 11% and 5% per field with and without material override. The underdose value doesn't change for the gantry angles investigated in this study, that is, from 5° to 30°. However, the underdosing values decrease from ∼11% to ∼8% per field as the depth increases from 2.0  to 5.5 cm.

The gamma pass rates of the two plans with and without the material override are both 100%, which indicates that it is not necessary to contour and assign a material to the hardware. Note that the diameter of the screw is overestimated in the contour, resulting in exaggerated effects in the plan with the material override in TPS. The gamma analysis results show that the delivered plans with the hardware are accurate within clinically acceptable tolerances,^22^ but we do note that the predicted dosimetric effect of the Ti‐screws in the studied planes is on par with, or is smaller than the 3% limit of the gamma evaluation. However, due to the high noise level in the film measurement, a more precise comparison between calculated and computed dose was not possible.

As shown in Figures 2d and 3, multiple fields with different gantry angles help to spread out the overdosed and underdosed regions, especially those that are relatively far from the hardware. At our institution, the minimum number of fields used to treat intracranial targets is 3. Assuming the hardware is in the beam path of all the three fields, at a depth of 3.5 cm from the screws, the maximum underdoses are 3.3% and 1.7% of the prescribed dose for the plans with and without the material override, respectively. For plans with large hinge angles between the coplanar or non‐coplanar fields, the underdose values are less than the above values if the hardware is not in the beam path of some fields. Figure 2d shows a screenshot of an axial view of a proton plan for a meningioma. The yellow arrows indicate the location of the titanium screws with a material override. The purple arrow indicates the region with < 2.0% under‐dose of the prescribed dose.

## 5 CONCLUSION

The two questions at the beginning of the manuscript have been answered. Based on the results of this study, a new standard operating procedure is implemented. The cranial titanium hardware is not contoured or assigned a material. The proton beam is allowed to pass through the hardware. The presence of the hardware in the beam path is not a factor for planners to consider when choosing between SFO and MFO.

## AUTHOR CONTRIBUTIONS

Peng Wang: perform the film analysis. Fionnbarr Timothy O'Grady: manuscript evaluation and protocol defining. Martin Janson: consulting on TPS performance and protocol defining. Daniel Kim and Kevin S. Choe: provide patients to be studied, confirming the hardware from surgeons and provide physician's opinion on the whole manuscript. MacLennan Grayden: provide help in planning. Caroline K. Bamberger: perform the delivery of the plans. Jiajin Fan: oversees the progress of the whole project.

## CONFLICT OF INTEREST STATEMENT

The authors declare no conflicts of interest.

## Supporting information

Supporting Information

## References

[1] Boehling NS , Grosshans DR , Bluett JB , et al. Dosimetric comparison of three‐dimensional conformal proton radiotherapy, intensity‐modulated proton therapy, and intensity‐modulated radiotherapy for treatment of pediatric craniopharyngiomas. Int J Radiat Oncol Biol Phys. 2012;82(2):643‐652. doi:10.1016/j.ijrobp.2010.11.027 21277111

[2] Park J , Park Y , Lee SU , Kim T , Choi YK , Kim JY . Differential dosimetric benefit of proton beam therapy over intensity modulated radiotherapy for a variety of targets in patients with intracranial germ cell tumors. Radiat Oncol. 2015;10(1):135. doi:10.1186/s13014-015-0441-5 26112360 PMC4480576

[3] Combs SE , Laperriere N , Brada M . Clinical controversies: proton radiation therapy for brain and skull base tumors. Semin Radiat Oncol. 2013;23(2):120‐126. doi:10.1016/j.semradonc.2012.11.011 23473689

[4] Fuss M , Hug EB , Schaefer RA , et al. Proton radiation therapy (PRT) for pediatric optic pathway gliomas: comparison with 3D planned conventional photons and a standard photon technique. Int J Radiat Oncol Biol Phys. 1999;45(5):1117‐1126. doi:10.1016/s0360-3016(99)00337-5 10613303

[5] Florijn MA , Sharfo AWM , Wiggenraad RGJ , et al. Lower doses to hippocampi and other brain structures for skull‐base meningiomas with intensity modulated proton therapy compared to photon therapy. Radiother Oncol J Eur Soc Ther Radiol Oncol. 2020;142:147‐153. doi:10.1016/j.radonc.2019.08.019 31522879

[6] Mash LE , Kahalley LS , Okcu MF , et al. Superior verbal learning and memory in pediatric brain tumor survivors treated with proton versus photon radiotherapy. Neuropsychology. 2023;37(2):204‐217. doi:10.1037/neu0000882 36480379 PMC10544942

[7] Gross JP , Powell S , Zelko F , et al. Improved neuropsychological outcomes following proton therapy relative to X‐ray therapy for pediatric brain tumor patients. Neuro‐Oncol. 2019;21(7):934‐943. doi:10.1093/neuonc/noz070 30997512 PMC6620628

[8] Child AE , Warren EA , Grosshans DR , et al. Long‐term cognitive and academic outcomes among pediatric brain tumor survivors treated with proton versus photon radiotherapy. Pediatr Blood Cancer. 2021;68(9):e29125. doi:10.1002/pbc.29125 34114294 PMC8316321

[9] Mash LE , Kahalley LS , Raghubar KP , et al. Cognitive sparing in proton versus photon radiotherapy for pediatric brain Tumor Is associated with white matter integrity: an exploratory study. Cancers. 2023;15(6):1844. doi:10.3390/cancers15061844 36980730 PMC10047305

[10] Stross WC , Malouff TD , Waddle MR , Miller RC , Peterson J , Trifiletti DM . Proton beam therapy utilization in adults with primary brain tumors in the United States. J Clin Neurosci Off J Neurosurg Soc Australas. 2020;75:112‐116. doi:10.1016/j.jocn.2020.03.011 32184042

[11] Patone H , Barker J , Roberge D . Effects of neurosurgical titanium mesh on radiation dose. Med Dosim. 2006;31(4):298‐301. doi:10.1016/j.meddos.2006.05.001 17134670

[12] Rakowski JT , Chin K , Mittal S . Effects of titanium mesh implant on dosimetry during Gamma Knife radiosurgery. J Appl Clin Med Phys. 2012;13(5):3833. doi:10.1120/jacmp.v13i5.3833 22955648 PMC5718236

[13] Sakamoto Y , Koike N , Takei H , Ohno M , Shigematsu N , Kishi K . Influence of backscatter radiation on cranial bone fixation devices. J Craniofac Surg. 2018;29(4):1094‐1096. doi:10.1097/SCS.0000000000004392 29498970

[14] Sakamoto Y , Koike N , Takei H , et al. Influence of backscatter radiation on cranial reconstruction implants. Br J Radiol. 2017;90(1070):20150537. doi:10.1259/bjr.20150537 27925774 PMC5685122

[15] Jabbari K , Rostampour M , Roayaei M . Monte Carlo simulation and film dosimetry for electron therapy in vicinity of a titanium mesh. J Appl Clin Med Phys. 2014;15(4):4649. doi:10.1120/jacmp.v15i4.4649 25207397 PMC5875510

[16] Tomida M , Kamomae T , Nishio T , Yanagi T . Dose perturbation effect of titanium implant in post‐operative proton therapy of head and neck cancer. Proceedings to the 59th Annual Conference of the Particle Therapy Cooperative Group, P089.

[17] Lin H , Ding X , Yin L , et al. The effects of titanium mesh on passive‐scattering proton dose. Phys Med Biol. 2014;59(10):N81‐N89. doi:10.1088/0031-9155/59/10/N81 24778368

[18] Zhu XR , Poenisch F , Li H , et al. A single‐field integrated boost treatment planning technique for spot scanning proton therapy. Radiat Oncol Lond Engl. 2014;9:202. doi:10.1186/1748-717X-9-202 PMC4262206 25212571

[19] Cubillos‐Mesías M , Baumann M , Troost EGC , et al. Impact of robust treatment planning on single‐ and multi‐field optimized plans for proton beam therapy of unilateral head and neck target volumes. Radiat Oncol. 2017;12(1):190. doi:10.1186/s13014-017-0931-8 29183377 PMC5706329

[20] Raystation 11A User Manual Chapter 7.2.

[21] Perles LA , Mirkovic D , Anand A , Titt U , Mohan R . LET dependence of the response of EBT2 films in proton dosimetry modeled as a bimolecular chemical reaction. Phys Med Biol. 2013;58(23):8477‐8491. doi:10.1088/0031-9155/58/23/8477 24240474

[22] Miften M , Olch A , Mihailidis D , et al. Tolerance limits and methodologies for IMRT measurement‐based verification QA : recommendations of AAPM Task Group No. 218. Med Phys. 2018;45(4). doi:10.1002/mp.12810 29443390

