# Supplementary material for: The impact of the titanium cranial hardware in proton single‐field uniform dose plans
Source: J Appl Clin Med Phys. 2024 Jun 12;25(9):e14374. doi: 10.1002/acm2.14374 (PMC11492323; doi:10.1002/acm2.14374)
Supplement: Supplementary file 1 — Supporting Information [file ACM2-25-e14374-s001.docx]

**Supplementary Appendix**

| Table 1 Management of Titanium Cranial Hardware Survey Results | | | | |
| --- | --- | --- | --- | --- |
|  | Proton system | TPS | Method |  |
| 1. | IBA Proteus | Raystation | Contour the screws and avoid proton beams passing through |  |
| 2. | Varian ProBeam | Eclipse | Totally ignore the hardware in planning |  |
| 3. | Hitachi PROBEAT | Eclipse | Contour and override the screws. Avoid proton beams passing through the long end of the screw. Evaluate the robustness to a 5% of range errors when proton beam passing through the long end of the screw. |  |
| 4 | IBA Proteus | Raystation | Contour and override the screws and allow proton beams passing through |  |
| 5. | IBA Proteus | Eclipse | Contour and override the screws and allow proton beams passing through the parts with less than 0.3 cm in length |  |
| 6. | Varian ProBeam | Eclipse | Contour and override the screws and allow proton beams passing through |  |
| 7. | IBA Proteus | Raystation | Totally ignore the hardware in planning |  |
| 8. | IBA Proteus | Raystation | Contour and override the screws. Try to avoid proton beams passing through, but it is not a must |  |
| 9. | Mevion S250i | Raystation | Contour the screws and avoid proton beams passing through |  |
